# Protective role of endorepellin in renal developmental programming

**DOI:** 10.3389/fcell.2022.929556

**Published:** 2022-10-18

**Authors:** Xiaoshan Tang, Manqing Sun, Qian Shen, Jia Rao, Xue Yang, Ye Fang, Tianchao Xiang, Shanshan Xue, Lei Sun, Hong Xu

**Affiliations:** ^1^ Department of Nephrology, Children’s Hospital of Fudan University, National Children’s Medical Center, Shanghai, China; ^2^ Shanghai Kidney Development and Pediatric Kidney Disease Research Center, Shanghai, China; ^3^ Institute of Developmental Biology and Molecular Medicine, Fudan University, Shanghai, China

**Keywords:** apopotosis, intrauterine growth restriction, renal development, nephron endowment, endorepellin

## Abstract

Adverse intrauterine and early postnatal environment cause reduced nephron endowment and subsequent hypertension, chronic kidney disease (CKD). Exploring modifiable approaches is particularly important to alleviate the global burden of CKD. Enhanced glomerular progenitor cell apoptosis is a major contributor to renal developmental programming. The differentially expressed protein perlecan, which we previously identified using proteomics, is an important extracellular matrix glycoprotein, and its domain V (endorepellin) can inhibit apoptosis through a paracrine form. In explanted mice embryonic metanephros, we found that endorepellin can rescue glomeruli-deficit phenotype resulting from malnutrition, and this protective effect was also verified *in vivo* using a renal developmental programming model which was given a low-protein diet during pregnancy. We further demonstrated that endorepellin significantly inhibited glomerular progenitor cell apoptosis which activates ERK1/2 phosphorylation. Our results show that endorepellin rescues the nephron number reduction in renal developmental programming, possibly through the inhibition of progenitor cell apoptosis *via* the ERK1/2 pathway.

## 1 Introduction

Chronic kidney disease (CKD) is a major public health problem globally (Collaboration, 2020). Accumulating evidence supports the relationship between adverse *in utero* or an early postnatal growth environment with the risk of CKD development in adult life (Hovi et al., 2007, Luyckx et al., 2013, Barker,2003, Gjerde et al., 2020a). Primary hypertension, the leading cause of CKD worldwide, has also been linked to low birth weight suggestive of intrauterine growth retardation (IUGR) (Ritz et al., 2011). The formation of the functional unit of the kidney, the nephrons, depends on the mutual induction between the ureteric bud and metanephric mesenchymal cells, which is extraordinarily sensitive to the effects of the adverse growth environment. Brenner proposed that reduced nephron endowment, defined as the nephron formation number at the completion of renal development, due to fetal malnutrition resulted in glomerular hyperfiltration, glomerulosclerosis, and further nephron loss, which led to hypertension and CKD. Congenital nephron deficit plays a central role in this pathological process known as “renal developmental programming,” which is supported by animal experiments and human data (Chen et al., 2010; Shen et al., 2011). Therefore, exploring rescue strategies for reduced nephron endowment in an adverse development environment can provide new pathways for preventing CKD.

Elevated glomerular progenitor cell apoptosis, i.e., metanephric mesenchymal cells, during kidney development has been implicated in nephron endowment reduction of IUGR. Maintenance and renewal of the metanephric mesenchymal cell population is dependent on inducing signals, growth factors, and other factors inhibiting apoptosis. Our previous comparative proteomic studies identified that perlecan was significantly decreased in the kidney development of the IUGR animal model and was associated with cell apoptosis (Welham et al., 2002; Li et al., 2010; Tafti et al., 2011). Perlecan, encoded by the *HSPG2* gene, is a heparan sulfate proteoglycan (HSPG) in the extracellular matrix around the metanephric mesenchyme and mainly produced by epithelial cells of the ureteric bud (Tang et al., 2016). Endorepellin is the C-terminal domain of perlecan and can be cleaved into a soluble form by using cathepsin L. Other cell and organ experiments have confirmed that endorepellins inhibit nearby cell apoptosis through the activation of ERK1/2-dependent anti-apoptotic pathways (Raymond et al., 2004; Laplante et al., 2006; Lee et al., 2011). This study investigated whether endorepellin supplementation increased nephron endowment and reduced cell apoptosis in renal developmental programming.

## 2 Materials and methods

### 2.1 *In vitro* model

The metanephros were isolated from E12.5 C57BL/6 mice embryos as described (Gjerde et al., 2020b) and cultured on a Transwell filter (0.4 μm pore size, CoStar) within individual wells of a six-well tissue culture dish containing 1,000 μl of Dulbecco’s modified Eagle’s medium/F12 media (Gbico) supplemented with 10% FBS. The kidneys were cultured at 37°C in air atmosphere containing 5% CO_2_ and 100% humidity for a total of 72 h (Barak and Boyle, 2011). 0.2% FBS was used during the last 24 h of culture to starve the kidneys, and 2ug/ml recombinant endorepellin (R&D Systems) was tested to protect the kidneys from programming. Whole-mount immunostaining of kidney explants was used to quantify the number of glomeruli and ureteric buds. Anti-E-cadherin (rabbit, 1:400 dilution, CST) and anti-Wt1 (rabbit, 1:200, Novus) were used as the markers of the ureteric branches and glomeruli, respectively. Alexa 594 anti-mouse (1:500, CST) and Alexa 488 ant-rabbit (1:500, CST) were used as secondary antibodies. Glomeruli and ureteric terminal tips were counted in the largest sagittal cross section from each kidney (Image J).

### 2.2 *In vivo* study

Virgin female C57BL/6 mice were used in this experiment. They were mated, and the appearance of vaginal plugs was designated as embryonic day 0 (E0). One group of dams was maintained on a normal-protein diet (NPD, 22% protein) throughout pregnancy. The other group of dams was placed on a low-protein diet (LPD, 6% protein) throughout gestation until delivery, when all mothers were fed standard rodent chow. The two diets were isocaloric; for detailed composition, refer to our previous report (Shen et al., 2011). LPD group newborns were randomly divided into the LPD+PBS group and LPD+DV group. Neonatal mice in the LPD+DV group were intraperitoneally administered 2ug/g recombinant endorepellin on the 1st, 3rd, and 5th days after birth, and the LPD+PBS group received the same volume of phosphate buffered saline (PBS). The endorepellin dose used is based on the previous *in vivo* regimen (Lee et al., 2011). The animal study protocols were approved by the Scientific and Ethics Committees at the Children’s Hospital of Fudan University (No. 2018021).

### 2.3 Glomerular counts

Kidneys were harvested at 4 weeks postnatal, and the acid maceration method was used for glomeruli number determination. A single right kidney was cut into 2mm or smaller pieces and incubated in 1 mmol/L hydrochloric acid for 30 min at 37°C. Acid was removed and replaced with 0.5 ml phosphate buffered saline (PBS; pH 7.4). The tissue was homogenized using a homogenizer. After low-speed centrifugation (3,000rmp, 10min), the supernatant was discarded and re-suspended in 10ml distilled water. A 20 µl sample was removed, placed on a slide, and overlaid with a cover slip. Using a ×10 objective lens, the number of glomeruli in the aliquot was counted. This was carried out thrice for each sample. The three results were averaged, and this value was used to determine the total number of glomeruli in the sample and, therefore, the kidney (Welham et al., 2002). The total number of glomeruli = average number×500.

### 2.4 Cell culture and apoptosis assessment

The metanephric mesenchyme cells (mK3 cells, Bluef Biotechnology) were cultured in DMEM (Gibco) supplemented with 10% fetal bovine serum (Gibco) and 1% penicillin/streptomycin (Invitrogen), incubated at 37°C, 5% CO_2_. During the last 24 h before flow cytometry, well-grown mK3 cells were incubated with a normal concentration of fetal bovine serum (10%FBS), serum deprivation (0.2%FBS), recombinant endorepellin (0.2%FBS+DV), and MEK1/2 inhibitor (U0126, Merck, Germany). The recombinant endorepellin concentrations of 5ug/ml were tested. The apoptosis of mK3 cells was assayed by flow cytometry with PE Annexin-V Apoptosis Detection Kit I (R&D Systems) according to the manufacturer’s recommendations.

### 2.5 Real-time PCR

Total RNA was extracted from metanephros cultured *in vitro* for 72 h and was transcribed to cDNA using the Prime Script RT Master Mix (TAKARA). Real-time PCR was performed using SYBR^®^ Premix Ex TaqTM (TAKARA) at the following conditions: 3 min at 95°C for one cycle, 2s at 95°C, and 20s at 60°C for 40 cycles. The gene levels were determined using the ΔΔCt method with GAPDH control. The primer sets included Hspg2: forward TGCTGCATACAGTGGTC TCC, reverse CCA​GGC​GTC​GGA​ACT​TGA​A; GAPDH: forward ACC​ACA​GTC​CAT​GCC​ATC​AC, reverse TCC​ACC​ACC​CTG​TTG​CTG​TA. All assays were run thrice.

### 2.6 Western blot

Each metanephro and mK3 cell per dish as one sample were extracted from the total protein and added with the phosphatase inhibitor. After protein concentration determination, 30 mg from each sample was separated in the gel and electrically transferred to the nitrocellulose membrane (Merck) after electrophoresis. The nitrocellulose membrane was blocked with 5% non-fat milk diluted in Tris-buffered saline plus 0.05% Tween-20 (TBST). After incubation overnight at 4°C with primary antibodies (anti-ERK1/2, CST; anti-pERK1/2, CST; anti-cleaved caspase-3, CST; and anti-GAPDH, Santa crus), horseradish peroxidase (HRP)-conjugated goat anti-rabbit IgG (Santa crus) was applied. The positive immune-reactive signal was visualized by using an enhanced chemiluminescence detection system with Immolbilon Western HRP Substrate (Merck, Billerica, United States). The expression level of the target proteins was normalized to GAPDH.

### 2.7 Histology experiment

The metanephroses of newborns or postnatal 3 days were carefully removed. The tissues were fixed in 4% paraformaldehyde and paraffin embedded. The tissues were sectioned at 4 μm, dewaxed, and rehydrated. Sections were stained with hematoxylin–eosin to observe morphological features. The sections were examined for molecular evidence of apoptosis using *in situ* end labeling (TMR or POD kit, Roche, Germany) according to the manufacturer’s instructions. Newborn metanephros sections were used to detect endorepellin expression level (anti-perlecan antibody, 1:100, Santa Cruz) by immunohistochemical staining. The MOD (mean optical density) of endorepellin in the nephrogenic zone was determined with Image-Pro Plus software.

### 2.7 Statistical analysis

Quantitative data were presented as the means ± standard deviations. Student’s t-test and non-parametric Mann–Whitney U-test were applied for statistical analysis of the differences between the two groups. The one-way ANOVA was applied to compare the differences among three or more groups followed by the Bonferroni method for post hoc comparisons. Differences with values of *p* < 0.05 were considered statistically significant.

## 3 Results

### 3.1 Endorepellin is downregulated in the intrauterine growth-retardation rodent model

**FIGURE 1:**
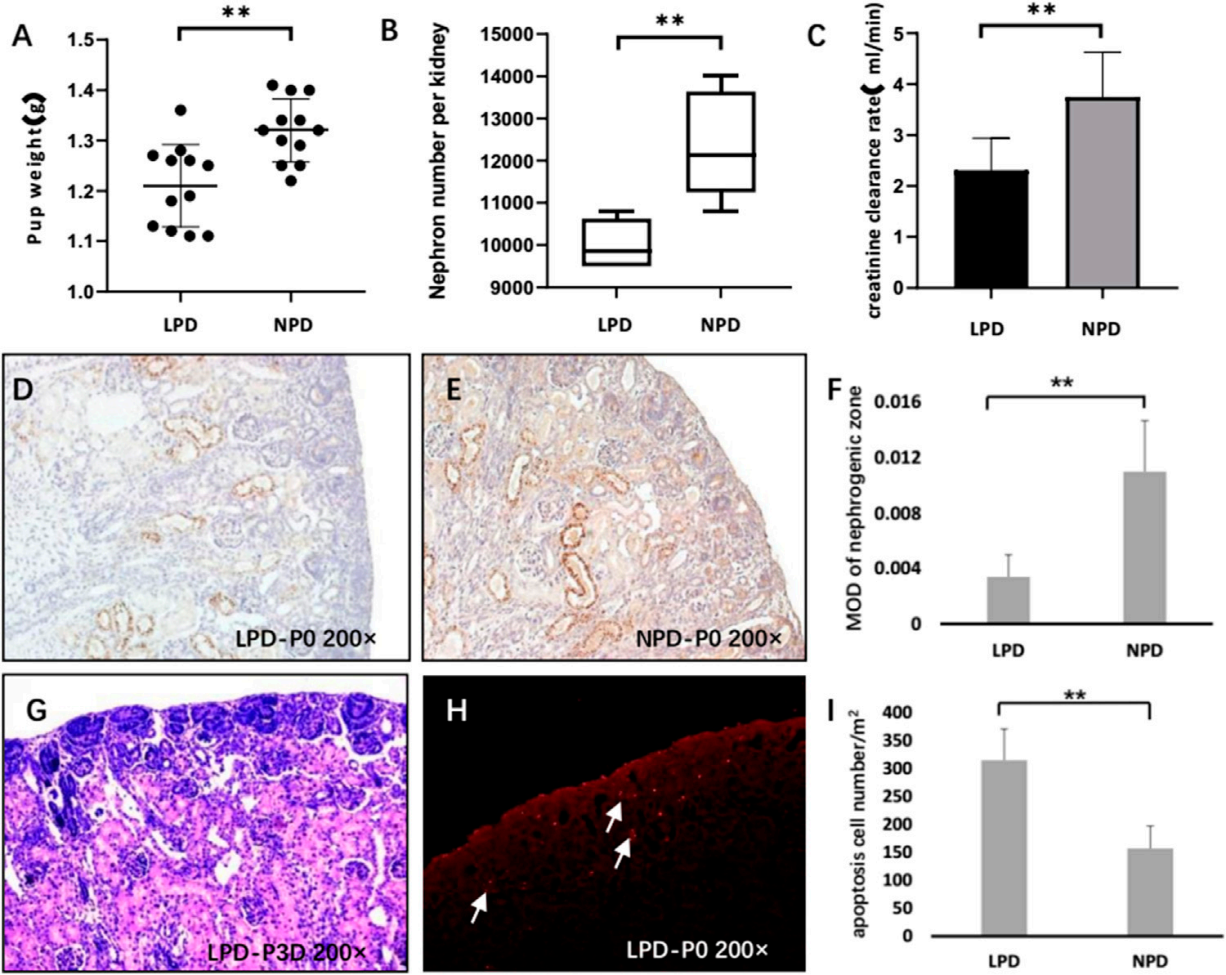
Endorepellin is downregulated in the IUGR rodent model. **(A)** The pup weight of the maternal low-protein diet (LPD) group at birth is significantly lower than that of the maternal normal-protein diet (NPD) group in mice (*n* = 12). **(B)** LPD reduces the nephron number in the kidney compared with NPD (*n* = 12). **(C)** After the 12-week follow-up, LPD mice showed decreased creatinine clearance rate (n = 8) **(D,E)** Representative kidney tissue sections stained with endorepellin at birth. **(F)** Densitometry analysis of DV immunohistochemistry in the nephrogenic zone as shown in D and E (*n* = 8). **(G)** HE staining showed that there were still many nephrons in the process of formation in 3-day postnatal mice. **(H)** Representative TUNEL image shows significant cell apoptosis in the nephrogenic zone of LPD newborn mice. **(I)** Apoptosis assessment of the nephrogenic zone in LPD and NPD newborn mice (*n* = 8). LPD, low-protein diet; NPD, normal-protein diet; DV, endorepellin; HE, hematoxylin–eosin staining; and TUNEL, TdT-mediated dUTP Nick-End Labeling. **p < 0.001.

Pregnant mice were given a low-protein diet (LPD, 6%) throughout pregnancy to successfully establish a mouse model of intrauterine growth retardation (IUGR). The body weight of newborn pups was significantly lower in the LPD group than in the normal-protein diet group (1.05 ± 0.081 vs. 1.32 ± 0.062 g, *p <* 0.01) (Figure 1A). After 12 weeks of follow-up, the low-protein diet during pregnancy significantly reduced the number of nephrons (9857 ± 556 vs. 12143 ± 1235, *p <* 0.01) and creatinine-clearance rate of offspring (2.31 ± 0.62 vs. 3.75 ± 0.88 ml/min, *p <* 0.01) (Figures 1B,C). Next, we used the immunohistochemical method to test whether the endorepellin expression in the nephrogenic zone of newborn was indeed reduced in the LPD group (0.0034 ± 0.0016 vs. 0.011 ± 0.0037, *p <* 0.05) (Figures 1D–F). Endorepellin is mainly distributed around the ureteric bud and in the matrix of mesenchymal cells in the nephrogenic zone. At the same time, we observed that active nephrons were still forming in mice 3 days after birth, and elevated apoptosis in the LPD group was detected ((315.18 ± 55.7 vs. 157.1 ± 40.32, *p <* 0.01) (Figures 1G–I). This suggests that the decrease in the number of nephrons in the LPD group may be related to the increase in progenitor cell apoptosis, and the late period of postnatal kidney development may provide a window for treatment.

### 3.2 Endorepellin-rescue nephron number *ex vivo* and *in vivo*

**FIGURE 2:**
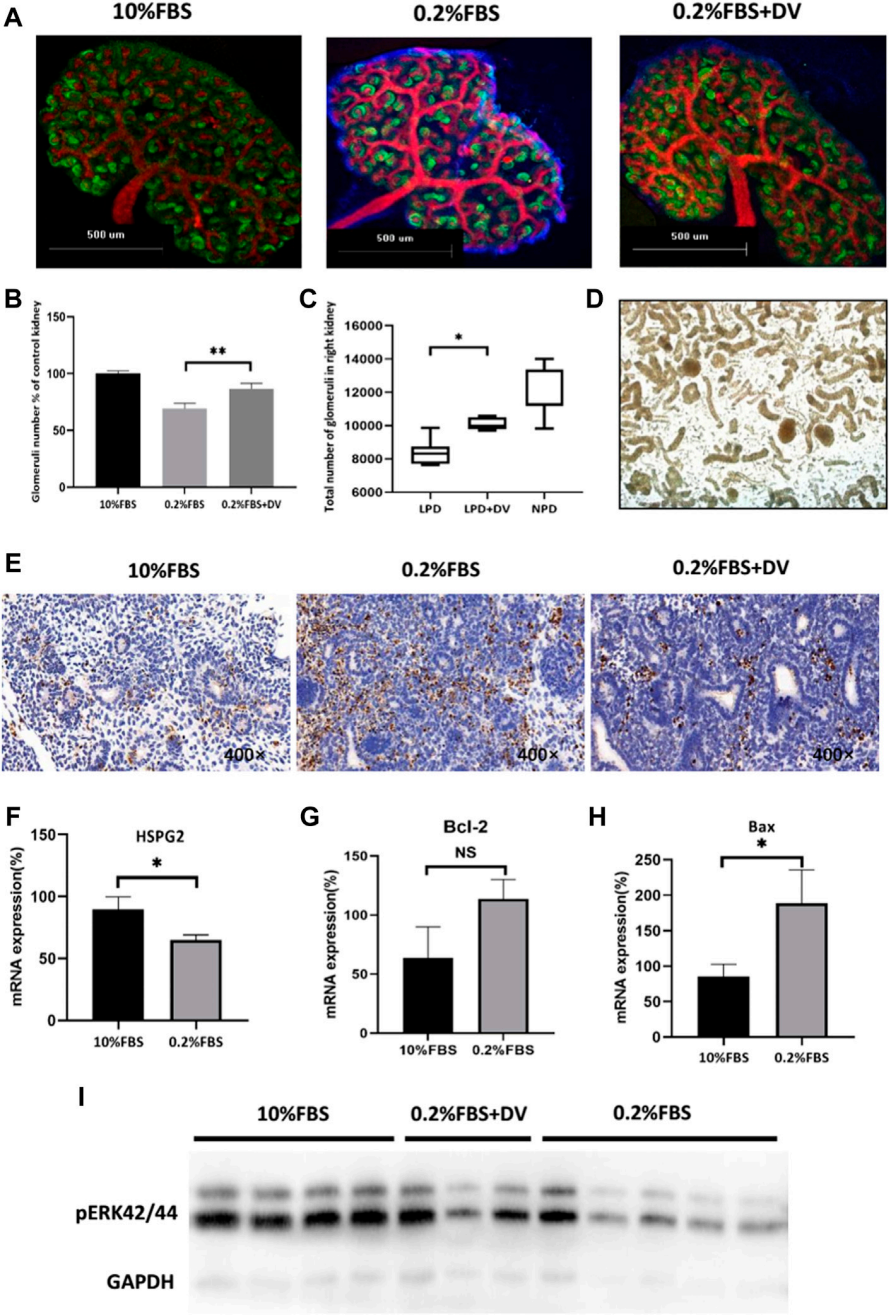
Endorepellin rescues the nephron number and reduces apoptosis in renal developmental programming. **(A)** Representative images of metanephros stained with Wt1 (green) and E-cadherin (red) antibodies to identify newly formed glomeruli and ureteric branches in normal serum conditions (10% FBS), serum deprivation (0.2% FBS), and co-culture with endorepellin (2ug/ml). **(B)** Serum deprivation in metanephros (*n* = 6) decreased the number of glomeruli to 69 + 4.2% of kidneys grown in 10% FBS (*n* = 6). Endorepellin significantly improved the number of glomeruli in serum-deprived metanephros to 86 ± 4.6% (*n* = 6). **(C)** The total number of glomeruli in 4-week-old mice exposed to a normal-protein diet (22%) or a low-protein diet (6%) with or without endorepellin during the first week of life. **(D)** Representative image of kidney-dissociated tissue under a microscope with hydrochloric acid digestion. Glomeruli are indicated by arrows. **(E)** Representative immunohistochemistry images of embryonic kidneys labeled with TUNEL (POD) to detect apoptotic cells. **(F–H)** Expression of Hspg2, Bcl-2, Bax, and mRNA in cultured metanephros exposed to 10% FBS or 0.2% serum deprivation, relative to control (*n* = 6). **(I)** Immunoblotting analysis of phosphorylated ERK1/2 in cultured metanephros exposed for 24 h to 10% FBS (*n* = 5), 0.2% FBS (*n* = 4), or 0.2% FBS supplemented with endorepellin (2ug/ml, *n* = 3). FBS, fetal bovine serum.

As we hypothesized, we determined whether endorepellin could rescue the phenotype of reduced nephron number against renal developmental programming in an adverse malnutrition environment. E12.5 metanephroses were either cultured under standard serum conditions (10% FBS) for the entire culture period or serum deprived (0.2% FBS) during the last 24 h of culture in the presence or absence of 2ug/ml endorepellin. After culture, the number of glomeruli was estimated by marker molecules of glomerular and ureteric bud epithelial cells (Figure 2A). There was no significant difference in the volume of kidneys deprived of 0.2% serum, but the number of glomeruli formed was significantly reduced (69 ± 4.2% of 10% FBS, *p < 0.001*), and this dysplasia can be rescued by endorepellin (86 ± 4.6% of 10% FBS group, *p < 0.001*) (Figure 2B).

To further validate the rescue effects of endorepellin, an *in vivo* renal programming model was used. Pregnant mice were fed an isocaloric low-protein (6%) diet throughout pregnancy, which resulted in reduction in the number of nephrons in the offspring compared with those having a normal-protein (22%) diet. Similar to the results of the *in vitro* model, an intraperitoneal injection of 2ug/g recombinant endorepellin (LPD+DV group) within 1 week after birth could significantly increase the number of nephrons (10,000 ± 1,300 vs. 8,322 ± 2,214, *p < 0.05*) (Figures 2C,D).

### 3.3 Endorepellin reduces apoptosis *ex vivo* and enhances the phosphorylation level of ERK1/2

In order to explore the potential mechanism by which endorepellin rescues the developmental programming of kidney, considering previous findings that cell apoptosis plays an important role in this process, we evaluated the apoptosis level of a cultured metanephros system. We found that apoptosis significantly reduced after adding endorepellin in the serum-deprivation *ex vivo* model (Figure 2E). We further examined the mRNA expression levels of *Hspg2*, *Bcl-2*, and *Bax* and the phosphorylation level of ERK1/2 in the *in vitro* model. We did find that serum deprivation reduced the mRNA expression level of *Hspg2*-encoding endorepellin in cultured embryonic kidneys (89.6 ± 8.8% vs. 65 ± 3.5%, *p < 0.05*) and significantly stimulated the expression of the pro-apoptotic factor *Bax* (85.5 ± 17.2% vs. 188.5 ± 47%, *p < 0.05*) but not *Bcl-2* (Figures 2F–H).

The ERK1/2 pathway was the key transducer of anti-apoptotic signals in various cell types. Studies have confirmed that endorepellin can participate in the process of anti-apoptosis by enhancing ERK1/2 phosphorylation in several types of cells (Raymond et al., 2004; Laplante et al., 2006; Lee et al., 2011). As the ERK1/2 pathway is the upstream pathway of bax, we tested whether this signaling pathway was involved in the response in the programming of the kidney. Decreased ERK1/2 phosphorylation was found in metanephros cultured with 0.2% FBS for 24 h, compared with 10% normal serum (Figure 2I). Again, serum-deprived metanephros exposed to endorepellin (2ug/ml) augmented ERK1/2 phosphorylation (Figure 2I). This result showed that endorepellin specifically induces ERK1/2 activation in serum-deprived metanephros and may reduce glomerular precursor apoptosis by enhancing the anti-apoptotic effect of ERK1/2-Bax.

### 3.4 Endorepellin protects from apoptosis in metanephric mesenchymal cells *via* the ERK1/2 pathway

**FIGURE 3:**
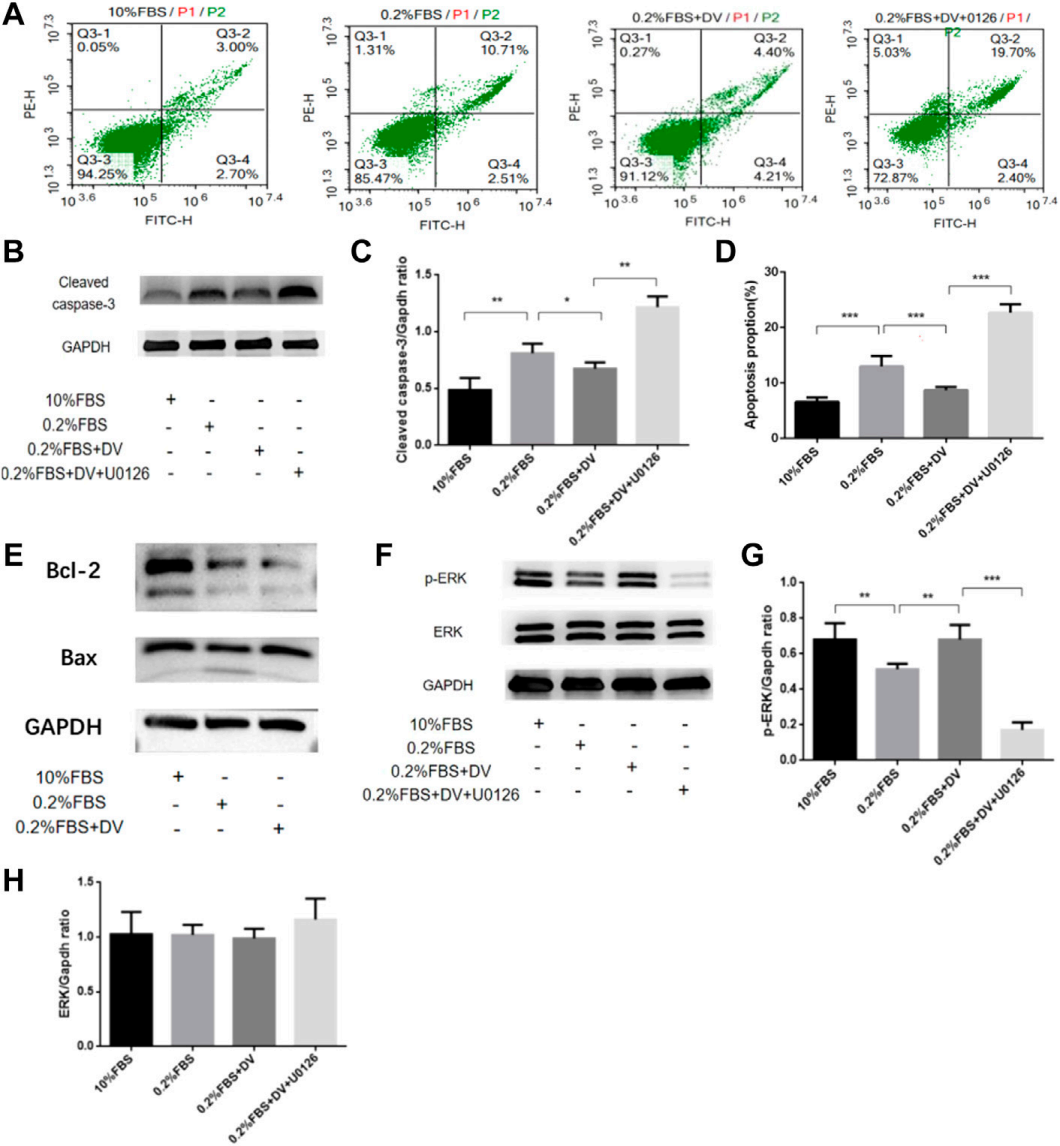
Endorepellin protects from apoptosis in the ureteric bud *via* the ERK1/2 pathway. **(A)** Annexin-V PE/FITC-stained apoptotic cells induced by serum deprivation and rescued by endorepellin in Mk3 cells. **(B)** Anti-cleaved caspase-3 western blot analysis of MK3 cells as labeled, with GAPDH as the internal loading control. **(C)** Densitometry analysis of cleaved caspase-3 western blot as shown in B as normalized to the corresponding GAPDH bands (*n* = 3). **(D)** Apoptosis ratios are shown in the diagram. Percentage of mK3 cells stained with Annexin-V PE/FITC after exposure for 24 h to normal serum (10% FBS), serum deprivation (0.2% FBS), serum deprivation supplemented with 5ug/ml endorepellin, or MAPKK inhibitor U0126 (*n* = 6); **(E)** apoptosis-related protein Bax and Bcl-2 expression were detected by western blotting. **(F)** Phospho-p44/42 MAPK and p44/42 MAPK western blot analysis of Mk3 cells as labeled, with GAPDH as the internal loading control. **(G,H)** Densitometry analysis of phospho-p44/42 MAPK and p44/42 MAPK using western blot as shown in F as normalized to the corresponding GAPDH bands *p* < 0.05, * *p* < 0.01, and * *p* < 0.001.

The aforementioned experiments suggest that endorepellin supplementation may play a protective role in rescuing the developmental programming of the kidney by activating the ERK1/2 pathway and inhibiting cell apoptosis. We next used the glomerular precursor cell line (metanephric mesenchymal cells, mK3 cells) to confirm this process. To identify whether serum deprivation can induce metanephric mesenchyme cell apoptosis, flow cytometry analyses by Annexin-V-FITC and PI double-staining assay were performed (Figure 3A). The cleaved caspase-3 western blot outcome further confirmed this effect (Figures 3B,C). Compared to the normal serum-cultured cells, apoptosis cell rates of 0.2% FBS significantly increased to 12.98% (12.98 ± 1.84% vs. 6.58 ± 0.78%, *p < 0.001*) and can be abolished by endorepellin supplementation (Figure 3D). The apoptosis cell rates decreased to 8.68 ± 0.56% with 5ug/ml endorepellin, and this effect was blocked by the MEK1/2 inhibitor (U0126) (Figure 3D).

Subsequently, to identify the underlying apoptotic inhibition mechanisms, we detected the expression levels of apoptosis-related proteins in mK3 cells. As shown in Figures 3E,F, serum deprivation resulted in the downregulation of Bcl-2, Bax, and ERK1/2 phosphorylation by western blotting. Adding endorepellin upregulates Bax, downregulates Bcl-2, and enhances ERK1/2 phosphorylation levels in mK3 cells, and this phenomenon also can be eliminated by using the MEK1/2 inhibitor (U0126) (Figures 3F–H). These findings suggested that endorepellin was able to inhibit ERK1/2-dependent cell apoptosis in mK3 cells.

## 4 Discussion

About 30 years ago, Brenner et al. proposed “renal developmental programming” that low birth weight might predispose for CKD and hypertension in adulthood through a mechanism of impaired nephron endowment in adverse *utero* environments (Brenner et al., 1988). Very little was found in the literature on modifiable correction methods for renal programming. This study suggests that endorepellin could rescue the nephron-reduction phenotype in *in vitro* and *in vivo* renal programming models and can inhibit the apoptosis of nephron-precursor cell mK3 through an ERK1/2-dependent mechanism. Given the high CKD burden globally, our results may provide innovative ideas for reducing the risk of CKD and ESRD during childhood in the future.

Previous studies strongly suggested the relationship between enhanced cell apoptosis in the metanephros and nephron reduction (Welham et al., 2002; Li et al., 2010; Tafti et al., 2011). Welham firstly reported that maternal low-protein diet increased mesenchymal cell apoptosis at the initial stage of metanephrogenesis (Welham et al., 2002). This was confirmed by subsequent studies that the nephron number reduction was abrogated by ouabain, which triggers a calcium-dependent nuclear factor-κB signal to inhibit cell apoptosis (Tafti et al., 2011). These could be explained by increased apoptosis in nephron progenitors during the active glomerular formation period resulting in a nephron deficit. Our previous work showed that there are massive amounts of glomerular development and elevated levels of cell apoptosis during the late gestational and early postnatal periods in IUGR rat (Tang et al., 2016). Interestingly, the differentially expressed endorepellin identified by comparative proteomics was significantly reduced in the mid and late stages of IUGR kidney development, and it was mainly expressed in the extracellular matrix of the metanephric cell and the periphery of the ureteric bud (Shen et al., 2011). This study further supports evidence from previous observations. Endorepellin has been suggested as an important endogenous inhibitor of apoptosis. We demonstrated that endorepellin supplementation rescued the nephron deficit in the renal developmental programming model through an ERK1/2-dependent mechanism of suppressing nephron-progenitor cell apoptosis.

The number of nephrons is determined by congenital genetic factors and an acquired intrauterine developmental environment (Charlton et al., 2021). In normal kidney development, metanephric mesenchymal cell (MMC) is the precursor of the glomerulus, and its maintenance including preventing apoptosis, cell proliferation, and trans-differentiation is the key determinants of nephron endowment (O'Brien, 2019). The ureteric bud can secrete GDNF-signaling molecules and other survival factors into the intercellular matrix to maintain MMC cell population balance (Li et al., 2021). Endorepellin is the main heparan sulfate proteoglycan (HSPG) in the extracellular matrix, composed of a core protein and several heparan sulfate (HS) side chains (Farach-Carson and Carson, 2007). Previous studies have focused on the HS side chains which can bind to growth factors such as GDNF, FGF, TGF-β, and EGF, act as growth factor reservoirs to mediate the interaction between epithelial cells and mesenchymal cells, and play a key role in organogenesis (Steer et al., 2004; Nigam and Bush, 2014). The current study is from the perspective of endorepellin (domain V of perlecan) to test its possible role in inhibiting the apoptosis of precursor cells in kidney development. Endorepellin has been shown to activate the ERK1/2 signaling pathway and, thus, inhibit apoptosis in other cell types. Soulez M et al. confirmed that this effect was dependent on endorepellin–β1-integrin interactions as β1-integrin blockade significantly reduces ERK1/2 activation. In addition, endorepellin has binding sites for growth factors such as EGF; EGF can activate the downstream ERK-signaling pathway after binding to its receptor. Endorepellin can be cleaved into the extracellular matrix by cathepsin L secreted by nearby apoptotic cells and inhibit the apoptosis of other cells through paracrine (Soulez et al., 2010). Here, we also confirm that endorepellin could inhibit metanephric mesenchymal cell apoptosis due to serum deprivation by Annexin-V PE/FITC-stained flow cytometry. Endorepellin rescued ERK1/2-Bax pathway inhibition in a dose-dependent manner. This phenomenon was also observed in the *in vivo* model.

The main weakness of this study is that the ERK1/2-Bax pathway-dependent apoptosis inhibition mechanism of the endorepellin-rescuing nephron reduction phenotype needs inhibitor validation to draw a definitive causal conclusion. The ERK1/2-Bax pathway is an important downstream pathway for various growth factors to regulate the proliferation of metanephric mesenchymal cells and ureteric bud epithelial cells. Our data show that endorepellin activates ERK1/2 phosphorylation levels and Bax protein expression levels *in vivo* and *in vitro*. Future experiments need to study the systemic effects of endorepellin in metanephric mesenchymal stem cells, including the receptor, transcriptional, and gene expression intervention mechanisms. In addition, this study lacks clinical research data to support the findings. Clinical studies have found that the concentration of endorepellin in urine is associated with the progression of kidney disease (Rocchetti et al., 2013; Surin et al., 2013). In future clinical studies, endorepellin levels in the urine of small-for-gestational-age or preterm infants can be compared with normal neonates, combined with other imaging assessments to develop a predictive model of renal function, and even clinical trials.

Collectively, we demonstrated in *in vivo* and *in vitro* models of programmed kidney development that endorepellin rescues the nephron number reduction, possibly through the inhibition of nephron-progenitor cell apoptosis by activation of the ERK1/2-Bax pathway. Our study proposed a new approach to correct nephron loss early in life.

## Data Availability

The original contributions presented in the study are included in the article/Supplementary Material; further inquiries can be directed to the corresponding author.
